# Gamma-Irradiated Influenza Virus Uniquely Induces IFN-I Mediated Lymphocyte Activation Independent of the TLR7/MyD88 Pathway

**DOI:** 10.1371/journal.pone.0025765

**Published:** 2011-10-05

**Authors:** Yoichi Furuya, Jennifer Chan, En-Chi Wan, Aulikki Koskinen, Kerrilyn R. Diener, John D. Hayball, Matthias Regner, Arno Müllbacher, Mohammed Alsharifi

**Affiliations:** 1 Department of Emerging Pathogens and Vaccines, The John Curtin School of Medical Research, Australian National University, Canberra, Australian Capital Territory, Australia; 2 Department of Microbiology and Immunology, School of Molecular and Biomedical Science, The University of Adelaide, Adelaide, South Australia, Australia; 3 Experimental Therapeutics Laboratory, Hanson Institute, Adelaide, South Australia, Australia; 4 Sansom Institute, The University of South Australia, Adelaide, South Australia, Australia; 5 Department of Medicine, The University of Adelaide, Adelaide, South Australia, Australia; Centers for Disease Control and Prevention, United States of America

## Abstract

**Background:**

We have shown previously in mice, that infection with live viruses, including influenza/A and Semliki Forest virus (SFV), induces systemic partial activation of lymphocytes, characterized by cell surface expression of CD69 and CD86, but not CD25. This partial lymphocytes activation is mediated by type-I interferons (IFN-I). Importantly, we have shown that γ-irradiated SFV does not induce IFN-I and the associated lymphocyte activation.

**Principal Findings:**

Here we report that, in contrast to SFV, γ-irradiated influenza A virus elicits partial lymphocyte activation *in vivo*. Furthermore, we show that when using influenza viruses inactivated by a variety of methods (UV, ionising radiation and formalin treatment), as well as commercially available influenza vaccines, only γ-irradiated influenza virus is able to trigger IFN-I-dependent partial lymphocyte activation in the absence of the TLR7/MyD88 signalling pathways.

**Conclusions:**

Our data suggest an important mechanism for the recognition of γ-irradiated influenza vaccine by cytosolic receptors, which correspond with the ability of γ-irradiated influenza virus to induce cross-reactive and cross-protective cytotoxic T cell responses.

## Introduction

We have reported previously, that live viral infections cause IFN-I dependent, generalized and systemic partial activation of lymphocytes, and this partial activation is characterized by elevated expression of the early activation marker CD69 and the co-stimulatory molecule CD86, but not the IL-2Rα chain, CD25 [1], [2]. We have found that the vast majority of lymphocytes undergo partial lymphocyte activation within 24h of infection with any of a large number of viruses from different virus families, with a return to base line levels at around day 5 post infection. In addition, we have reported that the magnitude of the IFN-I response and partial lymphocyte activation correlate with virus dose and virulence, and that γ-ray sterilized SFV failed to induce this phenomenon [1], [2].

It is well known that both Toll-Like Receptors (TLR) and cytosolic receptors (RIG-1 and MDA-5) are involved in recognition of viral RNA genomes and subsequently trigger IFN-I responses [3], [4]. Thirteen TLR have been identified in mammals and are expressed by macrophages, B cells, DC, T cells, fibroblasts and epithelial cells [5], [6]. The diversity of TLRs enables immune cells expressing them to survey the host environment for the presence of pathogens and each TLR binds a particular PAMP [3]. While interactions of some cell surface expressed TLRs with viral glycoproteins have been reported to signal the presence of cytomegalovirus [7] or respiratory syncytial virus [8], recognition of viral genomes by TLR3, 7, 8, and 9 within the endosomal compartment facilitates the detection of most viral infections [9]. It is currently accepted that dsRNA is recognised by TLR3, ssRNA is recognised by TLR7 and 8, and unmethylated 2’-deoxyribo (Cytidine-phosphate-guanosine) (CpG) DNA motifs present in bacterial and viral DNAs are recognised by TLR9 [9]. Upon recognition, homophilic Toll-Interleukin-I receptor (TIR) domains of the adaptor proteins (MyD88 or TRIF) interact to activate a cascade of proteins that result in IFN-I secretion [5]. Importantly, endosomal acidification and influenza ssRNA is required to induce TLR7/MyD88 dependent pathways in pDC [10], [11]. In addition to viral recognition by TLRs, two cytosolic viral RNA receptors have been identified, the caspase activation and recruitment domain (CARD)-containing RNA helicases retinoic acid inducible gene-1 (RIG-1) and melanoma differentiation antigen 5 (MDA-5) [4]. RIG-I and MDA-5 bind cytoplasmic uncapped 5’-triphosphate RNA and cytoplasmic dsRNA, respectively, and activate IFN-I gene expression via the IFN-β Promoter Stimulator 1 (IPS-1) adaptor protein [3], [4], [12], [13], [14]. It has been reported that plasmacytoid DC cells (pDCs) produce IFN-I in a TLR/MyD88 dependent and RIG-1 independent manner whereas myeloid DC cells (mDCs) are dependent on cytosolic receptors for viral recognition [15], [16].

In this study, we used inactivated influenza virus to investigate whether viral replication is an absolute requirement for partial lymphocyte activation to occur. We demonstrate that, unlike Semliki Forest virus (SFV), fully inactivated influenza virus is capable of inducing partial lymphocyte activation *in vivo*, regardless of the method used for inactivation. Furthermore, we used various gene knockout mice to illustrate that γ-irradiated influenza A virus (γ-flu), in contrast to other inactivated preparations, can induce lymphocyte activation in the absence of TLR7 pathways, which indicates recognition of inactivated virus by virus-specific cytosolic receptors.

## Materials and Methods

### Ethics statement

This study was carried out in strict accordance with the recommendations in the Guide for the Care and Use of Laboratory Animals of the Australian National University and The University of Adelaide. The protocol was approved by the Animal Ethics Committee of the Australian National University (Permit Number: JIG53.06) and the by the Animal Ethics Committee of The University of Adelaide (Permit Number: S-2010/099).

### Viruses and cells

Madin-Darby canine kidney (MDCK), baby hamster kidney (BHK), and T cell lymphomas (EL4, RMA, and transporter associated with antigen processing (TAP)-deficient (RMA-S)) cell lines were obtained from the American tissue culture collection (ATCC). Cells were grown and maintained in EMEM plus 5% FCS at 37°C in a humidified atmosphere with 5% CO_2_.

The influenza type A virus, A/PR/8 [A/Puerto Rico/8/34 (H1N1)] and A/PC [A/Port Chalmers/1/73 (H3N2)] was grown in 10-day-old embryonated chicken eggs (HiChick, South Australia). Each egg was injected with 0.1 ml normal saline containing 1 hemagglutination unit (HAU) of virus, incubated for 48 h at 37 ^o^C, and held at 4 °C overnight. The amniotic/allantoic fluids were then harvested, pooled and stored at −80 ^o^C. Viruses were purified using chicken red blood cells as previously described [17]. Briefly, infectious allantoic fluid is incubated with red blood cells for 45 mins at 4 °C allowing the viral-hemagglutinin to bind red blood cells, and then centrifuged to remove the allantoic fluid supernatant. The pellets were resuspended in normal saline, incubated for 1 h at 37 °C to release the virus from the red blood cells and then centrifuged to remove the red blood cells and collect the virus containing supernatant. Virus titres for A/PR8 and A/PC were determined by standard plaque assay on MDCK cells. Briefly, serial dilutions of virus stocks were assayed on MDCK cells monolayers in 6-well tissue culture plates. After 1 h adsorption, monolayers were overlaid with Eagle’s minimum essential medium with Earle’s salts medium (EMEM) containing 1.8% Bacto-Agar and incubated for 2–3 days. Cell monolayers were stained with 2.5% crystal violet solution and the plaques enumerated. Titres of purified stocks were 5×10^8^ PFU/ml (or 7×10^8^ TCID50/ml) for A/PC and 9×10^8^ PFU/ml (or 8.6×10^8^ TCID50/ml) for A/PR8.

The avirulent alphavirus, SFV was grown by infecting semi-confluent Baby Hamster Kidney (BHK) cell monolayers at a multiplicity of infection of 0.5 PFU per cell. Infected cells were incubated for 24 h, culture supernatants were harvested and centrifuged at 1200×*g* for 4 min and stored in single-use aliquots at −80 ^o^C. Viral titres were determined by plaque assay on Vero cells to be 10^7^ PFU/ml.

### Virus inactivation

For formalin inactivation, the viruses were incubated with 0.2% formalin at 4 °C for a week [18]. The formalin was then removed by pressure dialysis using normal saline for 24 h at 4 ^o^C. The dialysis method was adapted from Current Protocols in Immunology [19]. For UV inactivation, viruses were placed in 60-mm petri dishes with a fluid depth of 10 mm. The virus was exposed to 4000 ergs per cm^2^ for 45 mins at 4 ^o^C. For gamma ray inactivation, influenza viruses and aSFV received a dose of 10 kGy and 40 kGy respectively from a ^60^Co source (Australian Nuclear Science and Technology Organization – ANSTO). The virus stocks were kept frozen on dry ice during gamma irradiation. Sterility was tested by two independent methods: plaque assay using MDCK cells; and by inoculating embryonated eggs. The detection limit of our plaque assay is 10 PFU/ml and no plaque-forming unit was detected for the irradiated samples. These tests confirmed sterility in inactivated stocks. In addition, we have estimated the minimum inoculum required to cause a positive infection in embryonated eggs and found that the minimum egg-infectious dose that causes detectable HA titers in the allantoic fluid after 2 days of incubation is 0.1 PFU/egg. Embryonated eggs were inoculated with 100 μl of inactivated preparations per egg and incubated for 2 days at 37 °C and the allantoic fluid of individual eggs was harvested and tested for virus replication using HA assays. HA titers were negative in the allantoic fluid of these eggs, which illustrates a complete loss of virus infectivity in our inactivated preparations. The HAU titres of inactivated virus stocks were determined to be 7.29×10^4^ HAU/ml for γ-A/PR8, 2.43×10^4^ HAU/ml for formalin-A/PR8 and 8.1×10^3^ HAU/ml for UV-A/PR8.

CSL fluvax split vaccine 2008 is egg derived, β-propiolactone inactivated and contains 90 μg/ml of the hemagglutinin antigens of influenza A/Solomon Islands/3/2006 H1N1, A/Brisbane/10/2007 H3N2 and B/Florida/4/2006 (CSL Limited, Australia).

Solvay influvac subunit vaccine 2009 is egg derived, formaldehyde inactivated and contains 90 μg/ml of the hemagglutinin antigens of influenza A/Brisbane/59/2007 H1N1, A/Brisbane/10/2007 H3N2 and B/Brisbane/60/2008 (Solvay Biologicals LLC, Spain)

For *in vivo* studies, mice were injected intravenously (i.v) in the tail vein with 200 μl PBS containing either live or inactivated virus (2×10^7^ PFU equivalent) or 200 μl (18 μg HA) of the trivalent inactivated influenza vaccine (CSL fluvax or Solvay influvac).

### Hemagglutination assay

Live, inactivated virus and trivalent influenza vaccine preparations were serially diluted in a 100 μl volume on 96-well U-bottom microtiter plates. 0.5% chicken red blood cell suspensions were added to all wells and plates were incubated for 30 min on ice. This method was adapted from Current Protocols in Microbiology [20].

### Mice

C57BL/6, 129Sv/Ev (WTGR), MHC-II knockout (H2AB^−/−^) [21], interferon-type Iα receptor knockout (IFN-IR^−/−^) [22], interferon-γ receptor knockout (IFN-IIR^−/−^) [23], interferon type I and II double receptors knockout (IFN-I&IIR^−/−^), myeloid differentiation primary response gene 88 knockout (MyD88^−/−^) [24], Toll like receptor 3 (TLR3^−/−^) [25], Toll like receptor 7 (TLR7^−/−^) [26] mice were bred under specific pathogen-free conditions and supplied by the Animal Services Division at the John Curtin School of Medical Research, Canberra or the animal services at the University of Adelaide, Adelaide. 10∼14-week-old females were used in these experiments. Mice deficient in MyD88^−/−^, TLR3^−/−^, and TLR7^−/−^ were used with permission of Prof. S. Akira.

### ELISA assays for serum IFN-α, -β levels

Serum samples were collected from influenza or SFV immunized C57BL/6 mice at various time points post-immunization. IFN-α and -β levels in these serum samples were determined by ELISA. Briefly, Nunc-Immuno 96 Microwell plates were coated with monoclonal rat anti-mouse IFN-α (HyCult Biotechnology). Plates were then sequentially incubated with serum samples or a recombinant mouse IFN-α standard (HyCult Biotechnology) for 2 h at room temperature followed by a polyclonal rabbit anti-mouse IFN-α (PBL InterferonSource) for 2 h at rt and followed by horseradish peroxidase conjugated goat anti rabbit IgG (Sigma) for 2 h at rt and followed by peroxidase substrate (TMB substrate reagent set, Biosciences) for 30 mins. Optical density was measured at 450 nm. Serum IFN-α concentrations were estimated using a standard curve and expressed as Units/ml. One unit of mouse interferon alpha/beta is the amount of interferon alpha/beta which protects 50% of the indicator CHO cell population from viral induced cell death.

For IFN-β ELISA, monoclonal rat anti-mouse IFN-β was used (PBL InterferonSource) as capturing antibody and polyclonal rabbit anti-mouse IFN-β (PBL InterferonSource) as the detecting antibody. A recombinant mouse IFN-β (PBL InterferonSource) was used for establishment of a standard curve.

### Flow cytometric analysis

Spleens were harvested 1, 2 or 3 days post immunization and red blood cell depleted splenocytes were washed in PBS with 2% FCS. Fc receptors were blocked by incubation with mouse CD16/CD32 (Fcγ III/II receptor) Ab (BD Pharmingen) for 20 min at 4 ^o^C. Cells were washed and further incubated with a mixture of fluorescent-conjugated anti-CD3 (BD Pharmingen), anti-CD8 (BD Pharmingen), anti-CD19 (BD Pharmingen), anti-CD11c (BD Pharmingen), anti-CD25 (BD Pharmingen), anti-CD40 (abcam), anti-CD69 (BD Pharmingen), anti-CD80 (abcam) or anti-CD86 (BD Pharmingen) Abs. Dead cells were labelled with 7-aminoactinomycin D (Sigma-Aldrich). Stained cells were quantitated using a FACSCalibur (Becton Dickinson).

### ^51^Cr release assay

C57BL/6J mice were injected i.v with γ-irradiated A/PC. 7 days post-injection, spleens were harvested and red blood cell-depleted splenocytes were used as effector cells. Target cells were prepared by incubating EL4, RMA, and RMA-S T cell lymphomas with live or inactivated (γ-irradiated, formalin-inactivated, UV-inactivated) A/PC, followed by 1 h of incubation in medium containing 100 to 200 μCi of ^51^Cr. After three times washing, target cells were mixed with effector cells at different ratios and incubated for 8-h in a chromium release assay. The level of radioactivity in the supernatant was measured in a gamma counter. Specific lysis is given as mean percent lysis for triplicate wells, and values were calculated using the formula [(experimental cpm - spontaneous cpm)/(maximal release cpm - spontaneous cpm)] ×100.

## Results

### 1. Live and gamma-irradiated influenza viruses induce partial lymphocyte activation

**Figure 1:**
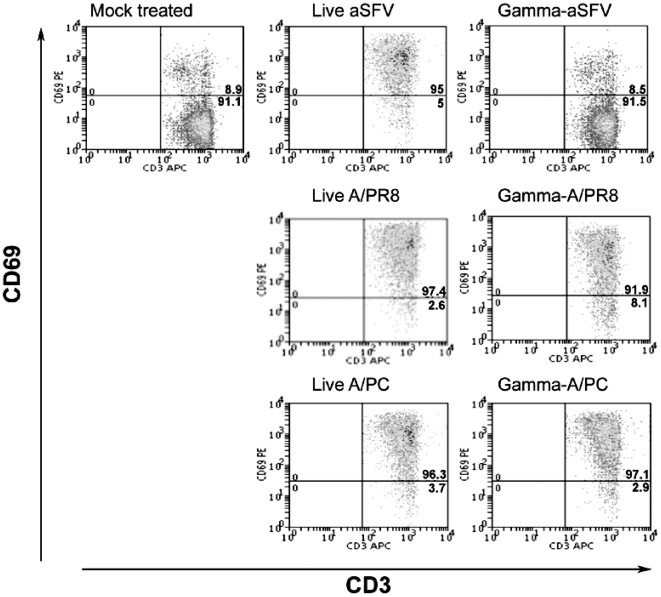
Gamma-irradiation renders SFV but not A/PR8 unable to induce partial lymphocyte activation. Mice were immunized i.v with SFV, A/PR8, or A/PC and their γ-irradiated counterparts. Splenocytes were harvested 24 h post immunization and cells were stained and analysed by FACS. Dot plots show CD69 expression levels on CD3^+^ splenocytes.

**Figure 2:**
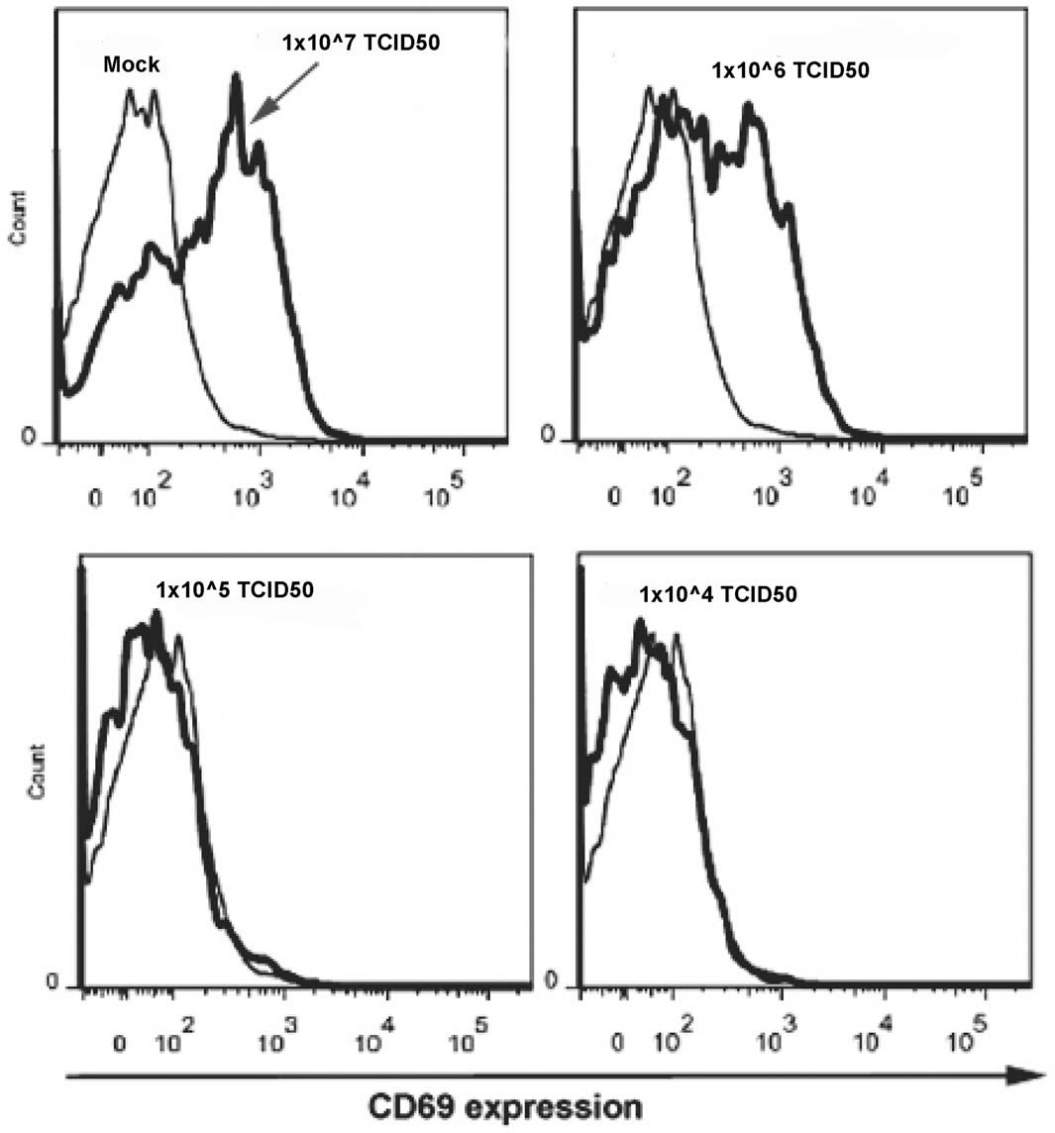
Generalized partial lymphocyte activation during A/PR8 infection is dose dependent. Splenocytes from wild-type mice were analysed for CD69 expression on CD3^+^ cells 24h following i.v injection of variable doses of A/PR8. Dot plots show fluorescence profiles of A/PR8 infected compared to naïve mice.

We have previously reported that for the alphavirus SFV, viral replication and subsequent IFN-I production is essential for the induction of partial activation of lymphocytes [2]. To test whether viral replication is a general requirement for systemic lymphocyte activation, we inactivated influenza A virus using γ-irradiation and compared the immune stimulatory ability *in vivo* with that of live and γ-irradiated SFV. Mice were injected i.v with SFV, A/PR8 or A/PC, or their γ-irradiated counterparts, and the cell surface expression of CD69, CD86, and CD25 was analyzed at days 1, 2 and 3 post-injections. Consistent with our previous report [2], γ-SFV failed to induce partial lymphocyte activation while both live influenza (A/PR8 and A/PC) and their γ-irradiated counterparts induced up-regulation of CD69 expression on CD3^+^ T cells (Figure 1). Expression profile of CD69 on CD19^+^ cells as well as CD86 on both CD3^+^ and CD19^+^ cells were similar to that of CD69 on CD3^+^ cells (data not shown). Consistent with our previously published work [2], the level of partial lymphocyte activation induced by live A/PR8 was dose dependent (Figure 2). Similarly, we found the overall levels of partial lymphocyte activation induced by live and inactivated preparations to be transient as the cell surface expression of the activation markers return to background levels by day 3 post-infection (Figure S1).

### 2. The ability of gamma-irradiated influenza virus to induce IFN-I response

**Figure 3:**
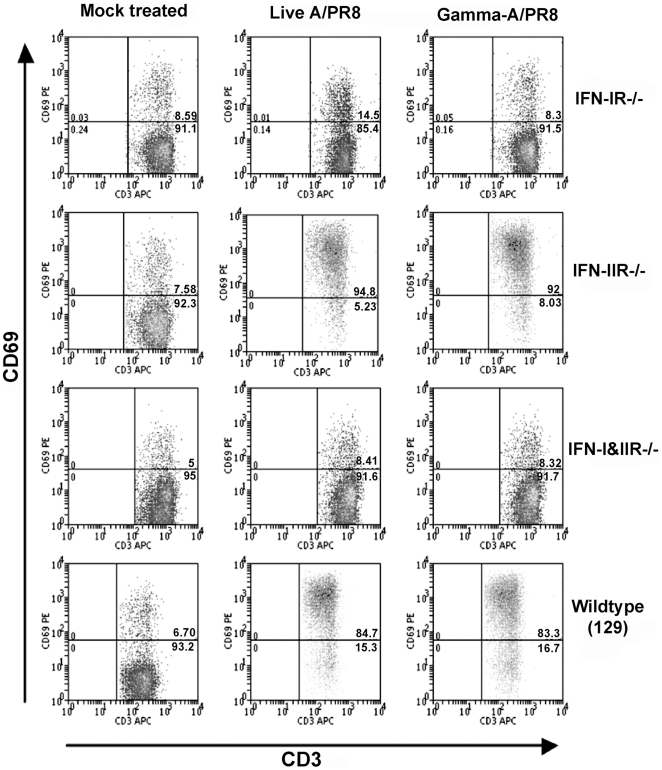
Generalized partial lymphocyte activation is dependent on an IFN-I response. Splenocytes from IFN-IR^−/−^, IFN-IIR^−/−^, IFN-I&IIR^−/−^ and wild-type 129 mice were analysed for CD69 expression on CD3^+^ cells 24h following *in vivo* immunization. Dot plots shows fluorescence profiles from mock, live A/PR8 infected and γ-A/PR8 immunized mice.

**Figure 4:**
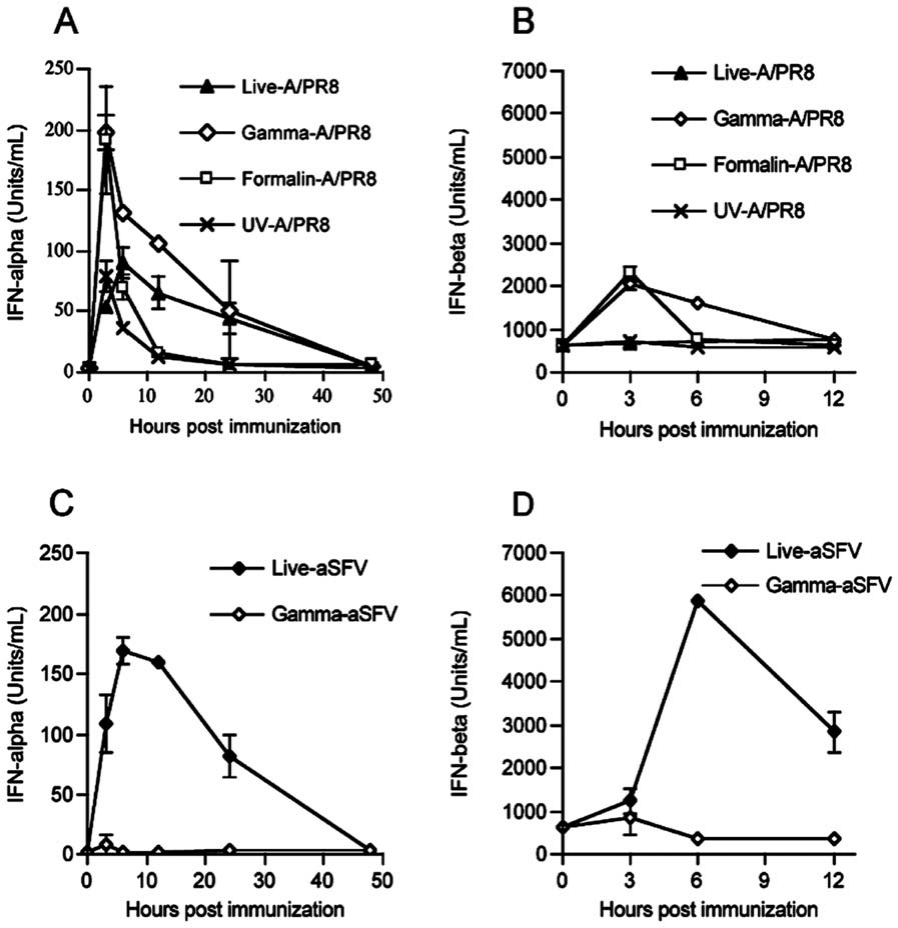
Comparison of IFN-α (A&C) and IFN-β(B&D) serum levels in mice immunized i.v with either live and or inactivated viruses. Mice were infected with SFV or A/PR8 or immunized with inactivated viruses (dose equivalent to 2×10^7^ PFU). Serum samples were tested for IFN-α and IFN-β concentrations and data expressed as mean (SD) Units/ml.

We addressed the question as to why γ-A/PR8, but not γ-SFV, retained its ability to stimulate partial lymphocyte activation after inactivation. We showed previously that systemic partial lymphocyte activation during acute viral infection requires IFN-I [1], [2]. Thus, we investigated lymphocyte activation in type I and/or type II IFN receptor(s) (IFN-IR^−/−^, IFN-IIR^−/−^, and IFN-I&IIR^−/−^) deficient mice following i.v administration of A/PR8 or γ-A/PR8. As shown in Figure 3, elevated expression of CD69 was only observed in wild-type 129 and IFN-IIR^−/−^ mice, but not in IFN-IR^−/−^ or IFN-I&IIR^−/−^ mice. These data confirm our previously published work regarding the role of IFN-I in lymphocyte activation. Consequently, we compared serum levels of IFN-α and IFN-β following i.v administration of live or γ-irradiated A/PR8 or SFV. As expected both live A/PR8 and SFV induced elevated IFN-α levels in sera, reaching peak values at 6 and 12 h, respectively, post infection and returned to background levels by day 2 (Figure 4). Elevated IFN-α levels induced by γ-A/PR8 peaked at 3 h post injection, and gradually declined to background levels by day 2 (Figure 4A). In contrast, no elevation of serum IFN-α was detected in mice injected with γ-SFV (Figure 4C). The peak IFN-α levels in mice treated with γ-A/PP8 were substantially higher than those seen with live A/PR8 suggesting a capacity of live replicating virus to interfere with IFN-α responses. This is concordant with the established IFN-I inhibitory role of the influenza NS1 protein [27], [28], which presumably would have to be produced in substantial amounts for significant interaction. In addition, our data show that live A/PR8 failed to increase IFN-β serum levels at any time point post infection (Figure 4B). Thus, differential induction of type I IFN-α and -β can be seen with both live and inactivated influenza virus preparations. In contrast, SFV induced IFN-β secretion with similar kinetics to that of IFN-α, reaching the peak serum level at 6 h post infection (Figure 4D). Importantly, γ-SFV failed to induce either IFN-α or IFN-β.

Bacterial endotoxin has been reported previously as a possible contaminant of egg grown viruses [29]. However, this is unlikely to contribute to the observed ability of γ-flu to induce IFN-I as we have routinely tested for bacterial contamination in our virus preparations. Nonetheless, in order to rule out this remote possibility, we tested cell surface expression of CD69 and CD86 on splenocytes from TLR2^−/−^ and TLR4^−/−^ mice following i.v injection of γ-A/PR8. As shown in Figure S2, both live and γ-A/PR8 preparations induced IFN-I mediated partial lymphocyte activation in TLR2^−/−^ and TLR4^−/−^ mice. Thus, the ability of γ-flu to induce IFN-I response, compare to γ-SFV, is not related to bacterial contamination.

### 3. The role of influenza viral glycoproteins

**Figure 5:**
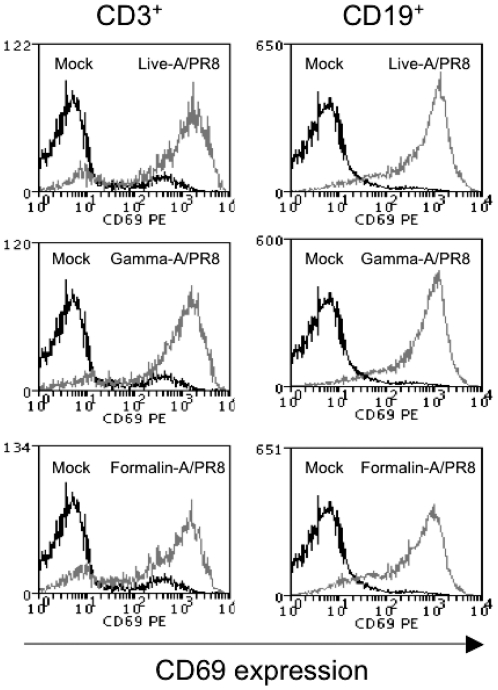
Generalized partial lymphocyte activation in MHC-II^**−/−**^ mice. Splenocytes from MHC-II^−/−^ mice were analysed for CD69 expression on CD3^+^ and CD19^+^ cells 24h after i.v injection of live or inactivated A/PR8.

Influenza virus is known as a lymphocyte mitogen [30]. The binding of the viral hemagglutinin to MHC-II molecules is believed to be responsible for influenza virus mitogenicity [31], [32], [33]. To investigate this possibility, mice defective in both α and β chains of MHC-II molecules (MHC-II^−/−^) were injected i.v with live, γ-irradiated or formalin-inactivated A/PR8. Splenocytes were harvested 24 h later, and cell surface expressions of CD86 and CD69 were analysed by FACS. An absence of MHC-II molecules did not influence the ability of inactivated viruses to induce CD69 (Figure 5) and CD86 (data not shown) expression on CD3^+^ or CD19^+^ cells.

**Figure 6:**
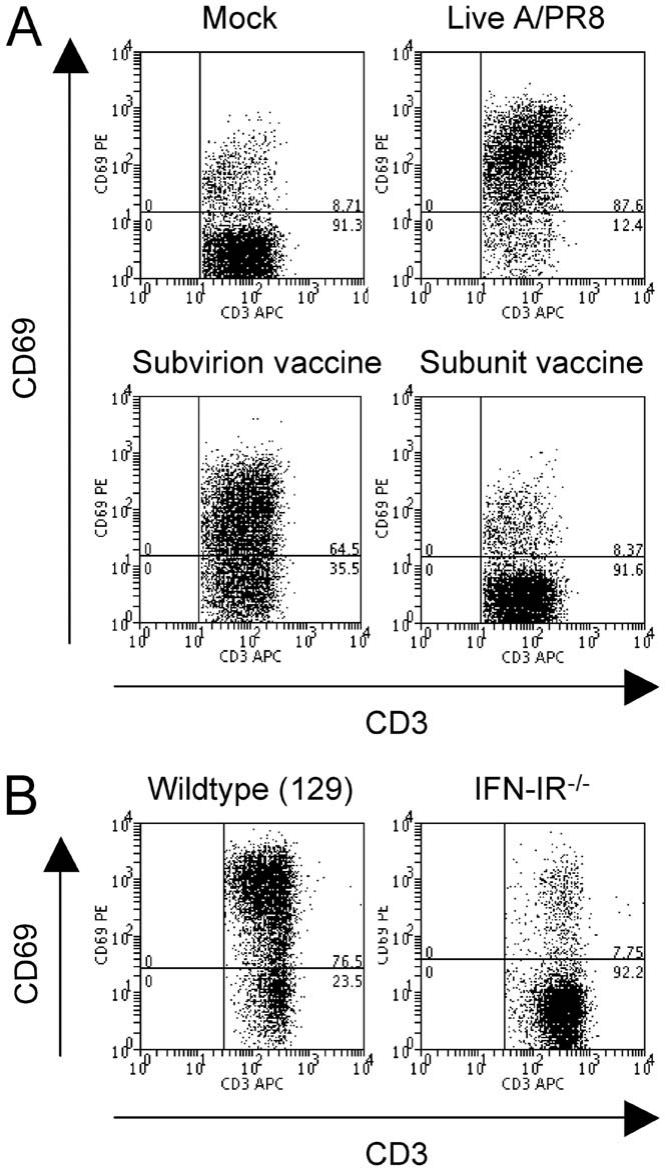
Subvirion, but not subunit, vaccine induces IFN-I dependent lymphocyte activation. A) Wild-type B6 mice were immunized i.v with either live (2×10^7^ PFU) or subvirion vaccine (CSL fluvax vaccine; A/Solomon Islands/3/2006 H1N1, A/Brisbane/10/2007 H3N2, B/Florida/4/2006; 18 μg hemagglutinin) or subunit vaccine containing purified HA and NA (A/Brisbane/59/2007 H1N1, A/Brisbane/10/2007 H3N2 and B/Brisbane/60/2008; 18 μg hemagglutinin). B) wild-type 129 and IFN-IR^−/−^ mice were immunized with subvirion vaccine. Splenocytes were harvested 24h post immunization and analysed for CD69 expression on CD3^+^ cells.

It has been reported previously that purified HA and NA (subunit vaccine) do not induce IFN-I response [34]. Therefore, to address a possible role of surface glycoproteins (HA and NA) in the observed *in vivo* lymphocyte activation, we immunized mice i.v with either of two different formulations of commercially available influenza vaccines: subvirion vaccine containing all the viral components (CSL fluvax vaccine; A/Solomon Islands/3/2006 H1N1, A/Brisbane/10/2007 H3N2, B/Florida/4/2006; 18 μg hemagglutinin) or subunit vaccine containing purified HA and NA (A/Brisbane/59/2007 H1N1, A/Brisbane/10/2007 H3N2 and B/Brisbane/60/2008; 18 μg hemagglutinin). Splenocytes were harvested 24 h post immunization and analysed for expression of activation markers. The magnitude of CD69 up-regulation induced by immunization with the subvirion vaccine was similar to that induced by live A/PR8 (Figure 6A). In contrast, the subunit vaccine did not induce up-regulation of either CD69 expression on CD3^+^ cells. The profile expression of CD86 was similar to that of CD69 (data not shown). To confirm the involvement of IFN-I in the induction of partial lymphocyte activation by the subvirion vaccine, IFN-IR^−/−^ and wild-type mice were injected i.v with the subvirion vaccine and their splenocytes were analyzed 24 h later. The subvirion vaccine elicited up-regulation of cell surface markers in wild-type129 but not in IFN-IR^−/−^ mice (Figure 6B). This provides evidence that the *in vivo* up-regulation of CD69 induced by trivalent influenza vaccine is mediated by IFN-I. In contrast, the subunit vaccine consisting of HA and NA viral antigens lacked this ability. Consequently, the mitogenic activity of surface glycoproteins has no or only a very limited role in the observed *in vivo* lymphocyte activation.

### 4. The role of TLRs in the induction of lymphocyte activation

**Figure 7:**
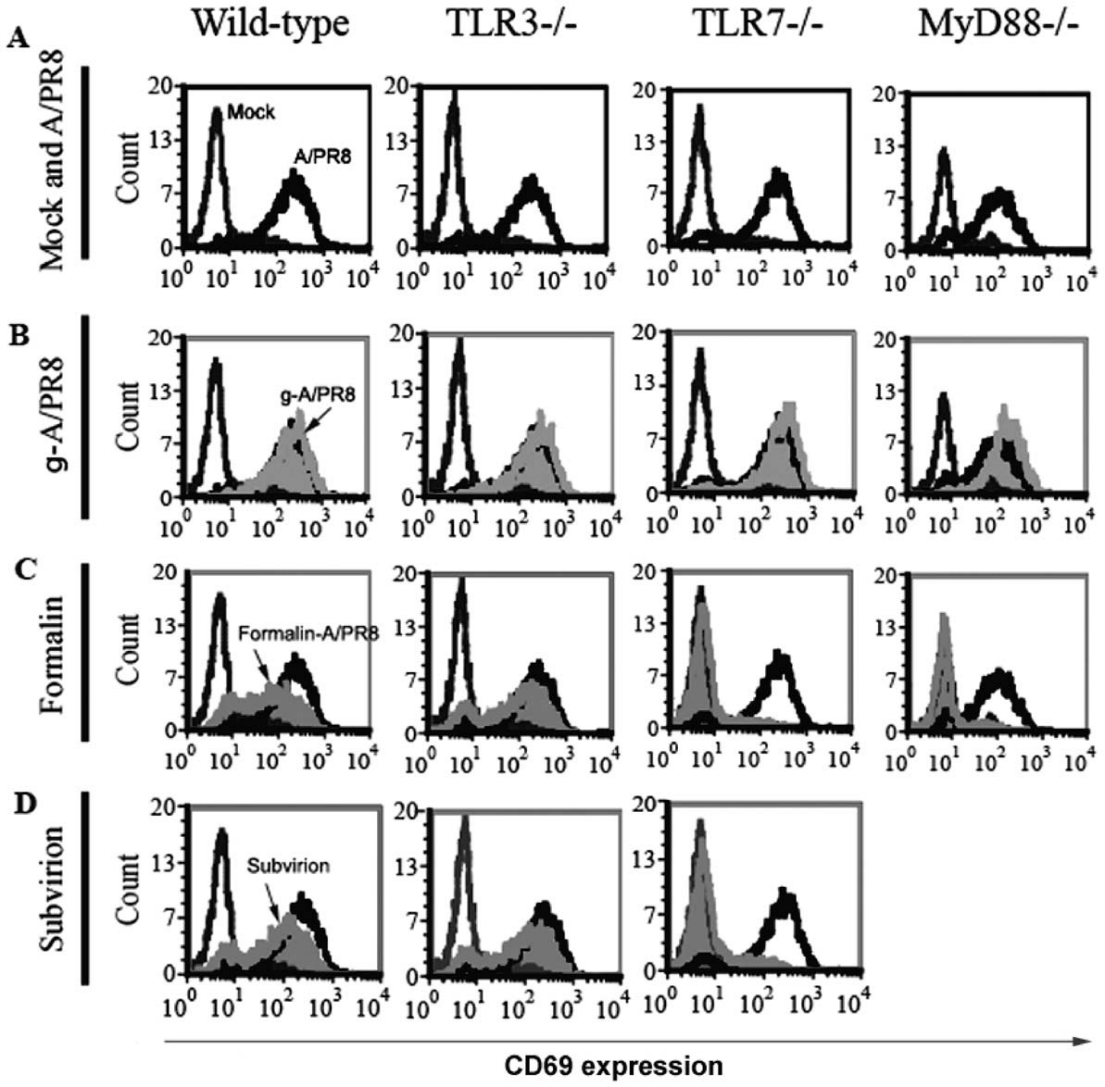
Gamma-irradiated influenza vaccine induces lymphocyte activation in the absence of TLR7/MyD88 signalling pathway. Splenocytes from wild-type 129, TLR3^−/−^, TLR7^−/−^ and MyD88^−/−^ mice were analysed for CD69 expression on CD19^+^ cells 24h following i.v injection of live A/PR8, gamma-irradiated-A/PR8, formalin inactivated A/PR8 and subvirion vaccine.

**Figure 8:**
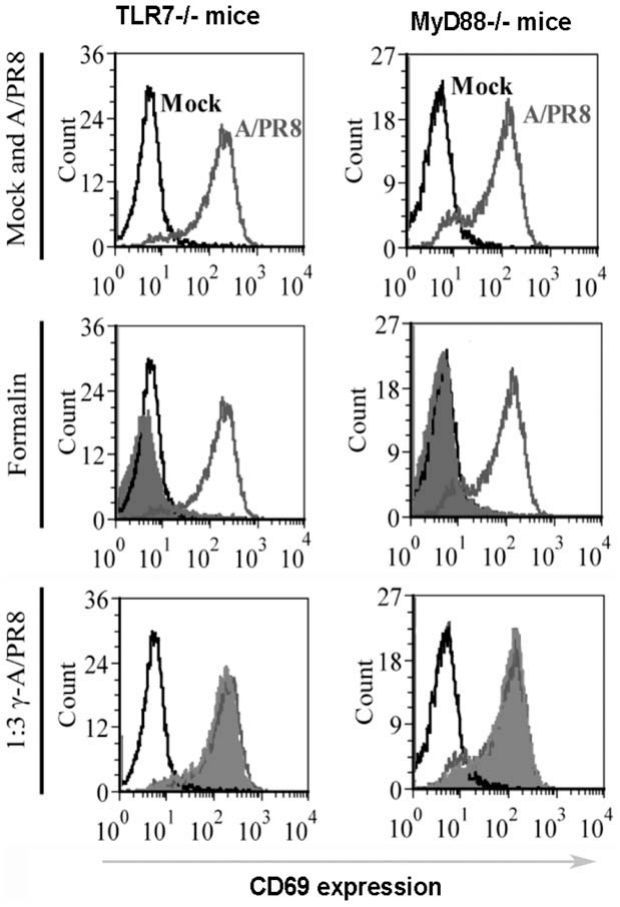
Gamma-irradiated influenza vaccine induces lymphocyte activation in the absence of TLR7/MyD88 signalling pathway. Splenocytes from wild-type 129, TLR7^−/−^ and MyD88^−/−^ mice were analysed for CD69 expression on CD19^+^cells 24h following i.v injection of live A/PR8, 1∶3 diluted gamma-irradiated-A/PR8, or un-diluted formalin inactivated A/PR8.

Gamma-irradiated viruses are expected to be able to fuse with the cell membrane, then releasing their contents into the cytoplasm in a similar manner to their ‘live’ counterparts. Since γ-ray inactivated viruses are incapable of replication, they can only be recognised by TLR7 and cytosolic receptors, consequently inducing IFN-I in MyD88-dependent or IPS-I-dependent pathways, respectively. In contrast, split (subvirion) vaccines consist of disrupted virus particles and therefore viral RNAs are present in these preparations and can only be recognised by TLR7 to induce IFN-I. In addition, formalin-inactivated viruses are prone to have rigid virion structure due to protein cross-linking [35], [36]. Similar to subvirion vaccine, formalin-inactivated influenza viruses are not expected to enter the host-cell cytosol and consequently their viral RNAs can only induce IFN-I via TLR7-dependent pathway. To test these predictions, we injected TLR3^−/−^, TLR7^−/−^, MyD88^−/−^ and wild-type B6 mice with the various inactivated influenza viruses and vaccine preparations and tested lymphocyte activation 24h later. As shown in Figure 7, splenocytes from all mouse strains (wild-type and knockouts) expressed high levels of CD69 on CD19^+^ cells following infection with live influenza virus. Similar data were observed for CD69 expression on CD3^+^ cells and also for CD86 expression on CD19^+^ and CD3^+^ cells (data not shown). Importantly, similar to live virus, splenocytes from all mouse strains showed high levels of CD69 expression following i.v injection of γ-A/PR8 (Figure 7B). In contrast, i.v administration of formalin-A/PR8 or split TIV vaccine (CSL vaccine) failed to induce lymphocyte activation in TLR7^−/−^ and MyD88^−/−^ mice (Figure 7C and D). Both preparations were, however, capable of inducing lymphocyte activation in wild-type and TLR3^−/−^ mice. Considering that formalin-inactivation affected the HAU quantity (3 fold reduction in HAU titres), we treated TLR7^−/−^, MyD88^−/−^ and wild-type B6 mice with either diluted γ-flu (1∶3 dilution) or undiluted formalin-inactivated vaccine preparations and tested lymphocyte activation 24h later. As shown in Figure 8, injection of live A/PR8 and diluted γ-A/PR8, in contrast to undiluted formalin-A/PR8, induced high levels of CD69 expression on CD19^+^ cells in wild-type and knockout mice. Similar data were observed for CD69 expression on CD3^+^ cells and also for CD86 expression on CD19^+^ and CD3^+^ cells (data not shown). Therefore, the inability of formalin-inactivated flu to induce IFN-I-dependent lymphocyte activation in TLR7^−/−^ and MyD88^−/−^ mice is not related to a reduced HA activity. Consequently, our data regarding the ability of γ-A/PR8 to induce IFN-I dependent lymphocyte activation in TLR7^−/−^ and MyD88^−/−^ mice must be related to the ability of γ-A/PR8 to deliver inactivated viral genomes and the associated viral proteins into the cytosol to be recognised by cytosolic receptors.

### 5. Sensitisation of target cells for lysis by Tc cells

**Figure 9:**
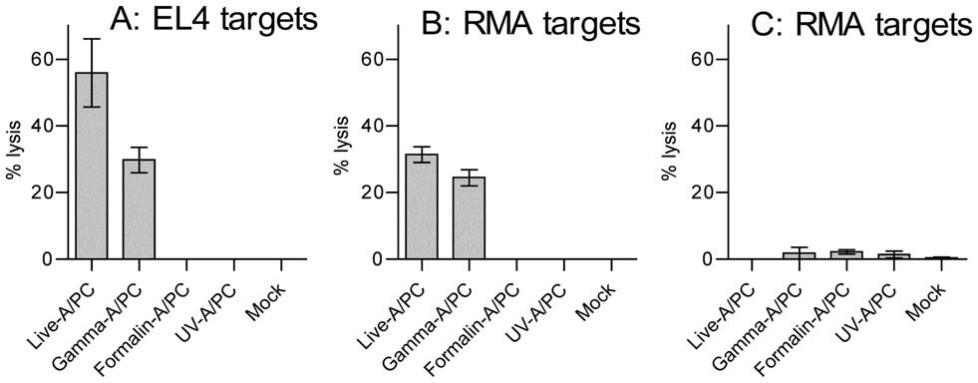
Target cell sensitization. EL4 (A), RMA (B) or RMA-S (C) cells were incubated with a multiplicity of infection of 1 PFU of live or 10 PFU-equivalent of inactivated (gamma-, formalin- or UV-) A/PC. These cells were used as targets in ^51^Cr release assay. Effectors cells were generated by i.v injection of C57BL/6 mice with gamma-inactivated A/PC (10^8^ PFU equivalent) and splenocytes harvested 7 days post-injection. Each bar represents the mean percentage ± S.D. Specific lysis values were interpolated from a regression curve at effector:target ratio of 40∶1.

We have previously illustrated the ability of γ-flu to induce cross-reactive and cross-protective Tc cell responses [37], [38], [39], [40]. It is well known that Tc cell responses are strictly regulated by TCR-mediated recognition of viral antigens presented in the context of MHC-I molecules. Therefore, in contrast to formalin-inactivated preparation, γ-flu must have the ability to deliver viral Ag into MHC-I presentation pathway. To test this, we investigated the ability of various inactivated influenza preparations to sensitise mouse-derived EL4, RMA, and RMA-S T cell lymphomas for lysis by Tc cells in vitro. Cells were incubated with a multiplicity of infection of 1 PFU/cell for live A/PC and 10 PFU-equivalent/cell of inactivated A/PC for 1 h. These treated cells were used as targets in ^51^Cr release assays to test the cytolytic activity of primary γ-A/PC immune Tc cells *in vitro* (Figure 9). Our data clearly show that both EL4 and RMA cell lines were sensitised by live A/PC and γ-A/PC to give significant lysis above mock treated control targets (P<0.05, student’s T test). In contrast, formalin- or UV-inactivated A/PC failed to sensitise both cell lines for Tc cell lysis. Importantly, the TAP-deficient T lymphoma RMA-S cells, which are deficient in MHC-I Ag presentation, are not sensitised by γ-flu compared to their wild-type RMA counterpart. Therefore, γ-flu, in contrast to UV or formalin inactivated influenza virus can deliver Ag into MHC-I presentation pathway.

## Discussion

We originally reported that infection of mice with live viruses from a range of virus families (pox-, flavi-, alpha- orthomyxo- and adenoviridae) results in rapid, systemic, but partial, lymphocyte activation, that is mediated by IFN-I [1], [2]. We had also previously shown that SFV, when inactivated by ionising radiation, lost the ability to induce IFN-I and consequently was unable to induce partial lymphocyte activation. Here we initially analysed the ability of inactivated influenza virus preparations to stimulate IFN-I-mediated partial lymphocyte activation *in vivo.* The difference between SFV and influenza virus in their ability to induce partial lymphocyte activation after γ-irradiation was surprising and raised questions about our earlier conclusions [2], that viral replication was necessary for the induction of this phenomenon.

We have reported previously that virus inactivation by γ-irradiation follows basic physical laws, including the concept of an ‘exponential low’, which means that there always exists a finite probability that an organism may survive, irrespective of the irradiation dose used [41]. This proviso, however, does not affect the outcome of our study as irradiated preparations were subjected to infectivity testing. The sterility of irradiated preparations was tested by plaque assay on MDCK cells (for influenza) and Vero cells (for SFV). The detection limit of our plaque assay is 10 PFU/ml and no plaques were detected for any irradiated sample used in our studies. Furthermore, we tested the sterility of irradiated influenza preparation in 10-day embryonated eggs and found no detectable HA titers in the allantoic fluid of the inoculated eggs. Furthermore, our present (Figure 2) and published [2] data clearly show that the level of lymphocyte activation 24 h post infection is dose dependent and requires infectious doses far in excess of our detection limit of 10 PFU/ml (or 2 PFU/mouse). Therefore, any residual virus infectivity can be ruled out to contribute to the observed partial lymphocyte activation.

Gamma-irradiation is known to generate nicks in the nucleic acid genome without affecting virion structure [41]. Consequently, γ-irradiated viruses are capable of fusion with cell membranes and release their contents into the cytoplasm. Our data clearly show that i.v administration of γ-flu, but not γ-SFV, induces both lymphocyte activation and IFN-I responses. It is important to consider that 1) both influenza and SFV are enveloped ssRNA viruses, 2) γ-ray inactivated viruses are unable to replicate and 3) systemic partial lymphocyte activation is IFN-I dependent [1], [2]. Therefore, the difference in the ability of irradiated viruses to induce lymphocyte activation may be related to their ability to directly interact with cDCs and/or pDCs. However, Hidmark AS and colleagues have reported that recombinant SFV can induce systemic IFN-I synthesis in wild-type and MyD88^−/−^ mice [42]. They have also shown that IFN-I production by mDCs cultures to be independent of viral replication, but dependent on IRF3. On the other hand, influenza A virus has been shown to infect DCs [43], [44]. Thus, both viruses appear to have the ability to interact directly with DCs. Furthermore, recent studies have proposed a genome-independent pathway of IFN-I induction, in which influenza glycoproteins are recognized as the primary stimuli triggering IFN-I production. For example, Miller and Anders [45] showed that influenza virus-infected fixed cells were able to induce IFN-I production *in vitro*. The authors argued that fixed influenza virus-infected cells may present arrays of viral glycoproteins on their cell surface that may interact with an as yet unidentified receptor on IFN-I producing cells. However, this possibility is ruled out by the inability of the purified surface glycoproteins (commercially available subunit vaccine) to induce IFN-I dependent lymphocyte activation. In addition, the crucial role of TLR7 in the induction of lymphocyte activation by subvirion and formalin-inactivated vaccine illustrates the need for viral genome recognition. Therefore, it is not obvious at present why γ-SFV, but not γ-Flu, fails to elicit IFN-I and lymphocyte activation in mice and further work is clearly needed to solve this discrepancy. Nonetheless, we focused on the recognition of inactivated influenza vaccines and the associated induction of IFN-I and lymphocyte activation. It is currently accepted that TLR7 and TLR3 mediate the endosomal recognition of viral ssRNA and dsRNA genomes and that they are preferentially expressed by pDCs and mDCs, respectively [46], [47]. In addition, 5’-triphosphate RNA can be recognised by RIG-I cytoplasmic receptors. Interestingly, a recent study investigating the immunogenicity of various inactivated influenza vaccines has reported that whole β-propiolactone inactivated H5N1 preparations, but not subunit vaccines, trigger IFN-I responses via TLR7 recognition of viral ssRNA [34]. We confirmed the role of TLR7 in the recognition of whole inactivated influenza vaccines. However, our data regarding the commercially obtained split vaccine clearly contradicts published findings related to lab-made split vaccines. Nonetheless, both studies illustrated the ability of inactivated influenza vaccines to induce IFN-I responses. Importantly, our data illustrate the ability of γ-irradiated influenza virus to induce partial lymphocyte activation in TLR7^−/−^ and MyD88^−/−^ mice, which indicates the possible recognition of the inactivated (nicked) viral genomes by cytosolic receptors. This must be associated with the delivery of structural internal viral proteins, including the nucleoprotein, source of the dominant peptide determinants, into the cytosol of APCs with a consequent Ag presentation via MHC-I and subsequent T cell priming. To confirm this possibility, we tested the ability of γ-flu to sensitise mouse-derived EL4, RMA, and RMA-S cells for lysis by influenza-immune Tc cells. Our data clearly illustrate the ability of γ-flu, in contrast to other inactivated preparations, to sensitise both EL4 and RMA, but not RMA-S, cells. It is important to note that RMA-S cells were originally selected from mutagenized RMA cells on the basis of low cell surface expression of class I molecules [48], and that RMA-S cells have been reported previously to be unable to present influenza virus nucleoprotein to H-2D^b^-restricted (C57BL/6J) Tc cells [49]. Therefore, our data regarding the ability of γ-flu to induce IFN-I dependent lymphocyte activation in TLR7^−/−^ and MyD88^−/−^ mice as well as the ability to sensitise RMA cells to lysis by influenza-immune Tc provides conclusive evidence of the ability of γ-flu to deliver internal viral proteins into the cytosol of APCs. In conclusion, data presented in this study provide an explanation as to why γ-ray inactivated, but not UV or formalin inactivated, influenza viruses induce cross-reactive Tc cell responses [39] with the potential to lead to the development of a cross-protective “universal” influenza vaccine [40], [50].

## Supporting Information

Figure S1 **Kinetics of partial lymphocyte activation of splenocytes from immunized mice.** C57BL/6 mice were injected i.v with either live (2×10^7^ PFU) or inactivated viruses (2×10^7^ PFU equivalent); live A/PR8, gamma-inactivated A/PR8, formalin-inactivated A/PR8 or UV-inactivated A/PR8 and mock treated (dotted line). Splenocytes were harvested at 1, 2 and 3 days post injection and analysed for cell surface expressions of CD69 and CD86 on CD3^+^ or CD19^+^ cells. Data presented as percentage of cells expressing the surface marker. Data represent the mean ± SD of two mice per group.
(TIF)

Figure S2 **Gamma-irradiated influenza virus induces lymphocyte activation in TLR4** ^**−/−**^ **and TLR2** ^**−/−**^ **mice.** Splenocytes from TLR4^−/−^ (A) and TLR2^−/−^ (B) were analysed for CD69 and CD86 expression on CD3^+^ and CD19^+^ cells following *in vivo* injection of γ-A/PR8. Dot plots shows fluorescence profiles of immunized mice and mock treated mice. Day 1 post immunization data are shown.
(TIFF)
