# Supplementary figures and images for: Gamma-Irradiated Influenza Virus Uniquely Induces IFN-I Mediated Lymphocyte Activation Independent of the TLR7/MyD88 Pathway
Source: PLoS One. 2011 Oct 5;6(10):e25765. doi: 10.1371/journal.pone.0025765 (PMC3187801; doi:10.1371/journal.pone.0025765)

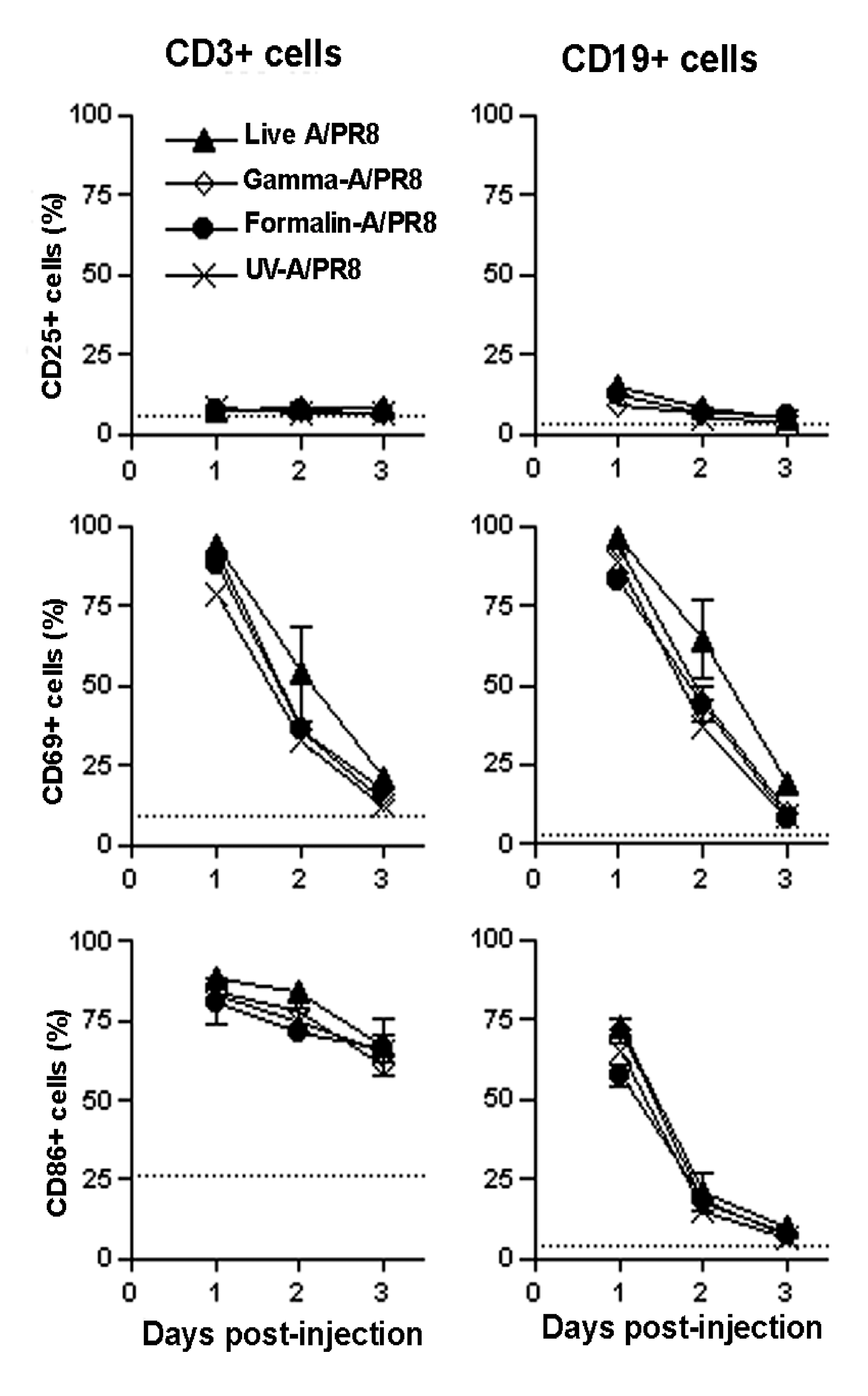

Supplement: Figure S1 — Kinetics of partial lymphocyte activation of splenocytes from immunized mice. C57BL/6 mice were injected i.v with either live (2×10^7^ PFU) or inactivated viruses (2×10^7^ PFU equivalent); live A/PR8, gamma-inactivated A/PR8, formalin-inactivated A/PR8 or UV-inactivated A/PR8 and mock treated (dotted line). Splenocytes were harvested at 1, 2 and 3 days post injection and analysed for cell surface expressions of CD69 and CD86 on CD3+ or CD19+ cells. Data presented as percentage of cells expressing the surface marker. Data represent the mean ± SD of two mice per group. (TIF) [file pone.0025765.s001.tif]

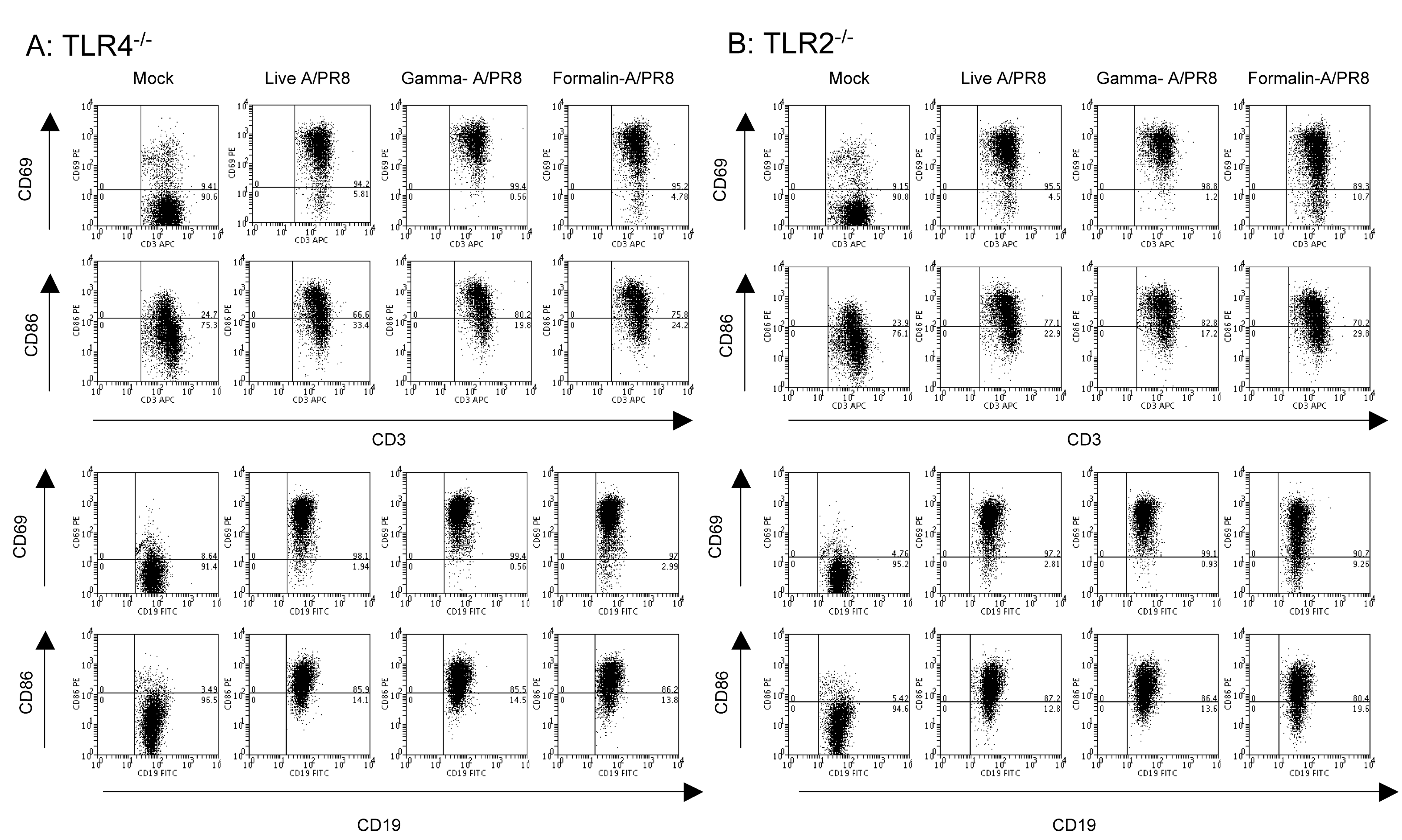

Supplement: Figure S2 — Gamma-irradiated influenza virus induces lymphocyte activation in TLR4 −/− and TLR2 −/− mice. Splenocytes from TLR4−/− (A) and TLR2−/− (B) were analysed for CD69 and CD86 expression on CD3+ and CD19+ cells following in vivo injection of γ-A/PR8. Dot plots shows fluorescence profiles of immunized mice and mock treated mice. Day 1 post immunization data are shown. (TIFF) [file pone.0025765.s002.tif]
